# Homologous equivalence study of immunogenicity after third dose of Covid-19 vaccine (recombinant) with an interval of six months after the second dose, comparing the interval of eight and 12 weeks between the first two doses

**DOI:** 10.1590/0074-02760240094

**Published:** 2024-09-09

**Authors:** Clarice Monteiro Vianna, Gloria Regina da Silva e Sá, Maria Vitória Hadland Seid, Luiz Antonio Bastos Camacho, Janaína Reis Xavier, Vitor Cardoso da Gama, Thalita da Matta de Castro, Ewerton Alves Portela dos Santos, Camila Dias de Almeida, Robson Leite de Souza Cruz, Marilda Siqueira, Maria de Lourdes de Sousa Maia, Clara Lucy de Vasconcellos Ferroco, Mia Ferreira de Araújo, Luis Fernando López Tort, Braulia Costa Caetano

**Affiliations:** 1 Fundação Oswaldo Cruz-Fiocruz, Instituto de Tecnologia em Imunobiológicos Bio-Manguinhos, Rio de Janeiro, RJ, Brasil; 2 Universidade Federal do Estado do Rio de Janeiro, Instituto de Saúde Coletiva, Rio de Janeiro, RJ, Brasil; 3 Fundação Oswaldo Cruz-Fiocruz, Escola Nacional de Saúde Pública, Rio de Janeiro, RJ, Brasil; 4 Fundação Oswaldo Cruz-Fiocruz, Instituto Oswaldo Cruz, Laboratório de Vírus Respiratórios, Exantemáticos, Enterovírus e Emergências Virais, Rio de Janeiro, RJ, Brasil; 5 Universidad de la República, Centro Universitario Regional Litoral Norte, Laboratório de Virologia Molecular, Salto, Uruguay

**Keywords:** Covid-19 vaccine, immunogenicity, equivalence study

## Abstract

**BACKGROUND:**

In response to the coronavirus disease 2019 (Covid-19) pandemic, Brazil authorised the Astra Zeneca/Fiocruz vaccine in January 2021. As the Delta variant emerged in May 2021, interval between vaccine doses was adjusted. By September 2021, the Brazilian National Immunisation Program recommended a booster dose for individuals over 70, and later expanded the recommendation to all adults.

**OBJECTIVES:**

Assess the equivalence of IgG antibody response against the Covid-19 S protein before and approximately 28 days after the third dose of a Covid-19 recombinant vaccine. Two groups received initial two doses with intervals of eight and 12 weeks.

**METHODS:**

This is a phase IV clinical study, uncontrolled, non-randomised. The study proposes calculating the ratio of geometric means titres (GMT) 28 days after the third dose, with a target ratio of confidence interval (CI) between 0.77 and 1.3.

**FINDINGS:**

In the primary endpoint, there was no equivalence between the eight- and 12-week intervals with a slight variation favouring the eight-week group. Post-third dose, both groups showed increases titres at 28 days, three months, six months and 12 months. Both groups responded similarly to Delta and Omicron BA.1, with a more significant increase for Delta.

**MAIN CONCLUSIONS:**

The study showed strong and consistent immune response in all age groups receiving the Covid-19 recombinant vaccine. Third dose elicited an increase in GMT by at least three times aligned with Ministry of Health strategies emphasising Bio-Manguinhos crucial role in pandemic control in the country.

The World Health Organization (WHO) officially declared the onset of the coronavirus disease 2019 (Covid-19) pandemic in March 2020. In Brazil, the licensing of two vaccines occurred in January 2021. The Astra Zeneca/Fiocruz vaccine, administered in a 0.5 mL dosage, exhibited a commendable overall efficacy, particularly in avoiding severe cases. Safety assessments were founded on data reported from a cohort of 12.282 vaccinated individuals, showing mild to moderate adverse reactions. The pre-registration efficacy stood 66.7%, and immunogenicity studies revealed a seroconversion rate exceeding 98% after the initial dose.^1^

In May 2021, the emergence of the Delta variant prompted certain countries to curtail the interval between vaccine doses.^2^ Subsequently, in September 2021, the Ministry of Health in Brazil recommended a supplementary booster dose for individuals over 70 years old, to be administered 28 days following the completion of the primary vaccination schedule.^3^ By November, the availability of booster doses was extended to encompass all individuals aged 18 and older.^4^

The Ministry of Health advised a preference for mRNA vaccines or, alternatively, viral vector vaccines for booster doses. In December 2021, the recommended interval for the booster dose was reduced to four months after the conclusion of the primary vaccination series.^5^ Studies suggested that the immune response decreases over a six-month period with intervals between vaccine doses of eight-12 weeks compared to those of 15-25 weeks.^6^ Nevertheless, even up to 200 days after the second dose, IgG Anti-S antibodies persist at levels surpassing those observed prior to the second dose, despite lower with intervals of eight-12 weeks compared to those of 15-25 weeks.

The same study investigated the immunogenic response of 75 participants who received a third dose of the ChAdOx1 nCoV-19 vaccine 28 days after their second dose. Results suggest a heightened immunogenic response compared to the second dose, with increased levels of neutralising antibodies against the alpha, beta and delta variants 28 days post the third dose.

As the end of December 2023, 518.977.927 doses of vaccines against Covid-19 were administered in Brazil. Notably, the Covid-19 AstraZeneca/Fiocruz vaccine contributed to around 28% of this overall vaccination effort.^7^ This article presents an assessment of the immunologic IgG antibody response against the Covid-19 S protein before and approximately 28 days, 90 days, 180 days and one year after the booster dose of a Covid-19 recombinant vaccine.

## MATERIALS AND METHODS

*Study design* - This is a phase IV clinical study, uncontrolled, non-randomised, only blinded to the laboratory technicians, stratified into two groups based on the interval between the first two doses (eight and 12 weeks), homologous. The main hypothesis was that a third dose of the Covid-19 AstraZeneca/Fiocruz vaccine (recombinant) administered four to eight months after the second dose is safe and equivalent in terms of immunogenicity when comparing the interval between the first two doses of eight weeks with the interval of 12 weeks. The secondary objectives of study were: assess the immunity in terms of geometric means titres (GMT) for both vaccine groups at three, six and 12 months after the administration of the booster; stratify immunogenicity equivalence by age group (< 40 years, 40 to 60 years, > 60 years); perform plate-based neutralisation antibody tests on a subset of 50 participants from both interval groups, pre-vaccination and 28 days of the third dose.

The study enrolled individuals of both sexes aged 18 years or above, who had received two doses of the Covid-19 (recombinant) vaccine, with an interval of eight or 12 weeks between the two doses, and a booster dose of Covid-19 (recombinant) vaccine, four to eight months after the second dose. The vaccination according to the intervals was by spontaneous demand and the recruitment of participants occurred from November 2021 to May 2022. Exclusion criteria encompassed individuals with contraindications to vaccination, those who were pregnant, using immunosuppressive medications, experiencing suspected symptoms or testing positive for Covid-19, previous vaccination with other Covid-19 vaccines (heterologous scheme) for the first and/or second dose, receiving other vaccine 28 days before study enrolment. After being included in the study, participants should be excluded if they received another Covid-19 vaccine other than the fourth dose, or if they received any vaccine 28 days after the third dose Covid-19, except for the influenza vaccine.

To enhance participant adherence, the study employed strategies such as reinforcing the need to adhere to the study protocol at each visit, making periodic calls for appointment confirmation, addressing questions during operating hours, providing support for adverse events, and assuring test result delivery. In case of missed visits, researchers attempted phone contact, escalating to a home visit after two unsuccessful attempts on consecutive business days.

**Fig. 1:**
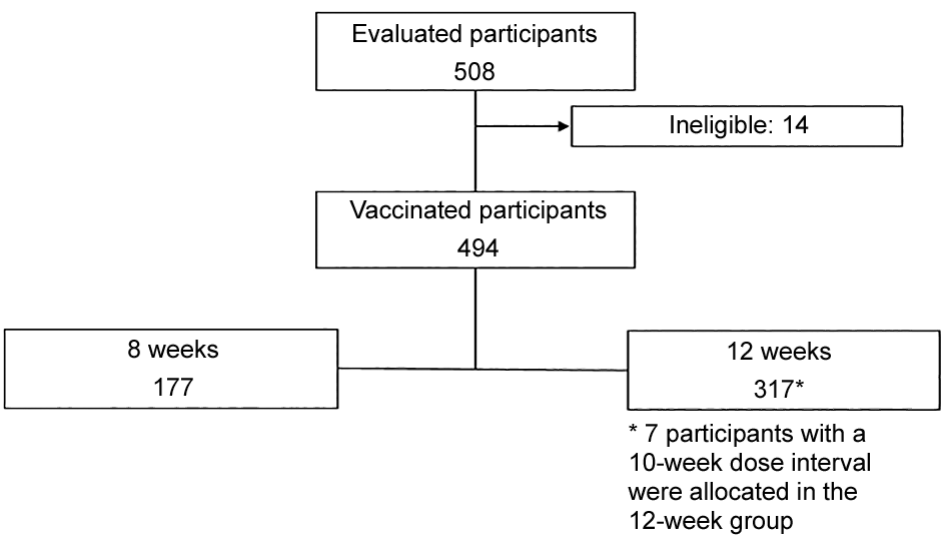
immunogenicity cohort analysis with laboratory data related to the outcomes of interest.

The cohort for immunogenicity analysis consisted of 494 vaccinated subjects, with 177 participants in the eight-week group and 317 in the 12-week group (Fig. 1). Of this total 233 participants had visits conducted within the limits for the anticipated window (maximum of 14 days), but considering the pandemic challenges for the visits, these 494 cohort had availably laboratory data related to the outcomes of interest and composed the cohort for the outcomes analysis of immunogenicity.

**Fig. 2:**
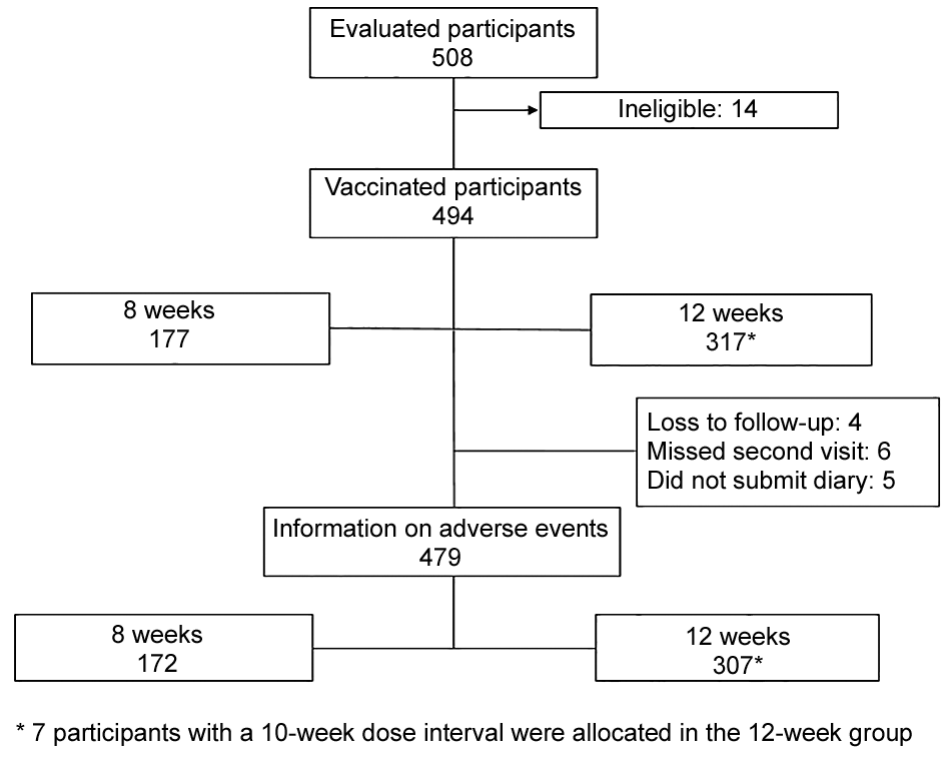
reatogenicity cohort analysis with information on post-vaccination adverse events.

The cohort for reactogenicity analysis included 479 vaccinated subjects for whom there was information on post-vaccination adverse events. There were 172 participants in the eight-week group and 307 in the 12-week group (Fig. 2).

*Laboratory methods* - For the evaluation of severe acute respiratory syndrome coronavirus 2 (SARS-Cov-2) IgG antibody levels for the main analysis of the study, serum samples were tested using a commercial kit, by chemiluminescent platform. The assay is a chemiluminescent microparticle immunoassay for qualitative and quantitative detection of IgG in human serum or plasma against the receptor-binding domain (RBD) of the SARS-CoV-2 spike protein. The platform requires a minimum of 100 µL of serum or plasma. Results releases by the equipment were used in the analyses. The test is a qualitative and quantitative test that detected IgG class antibodies, including neutralising antibodies, against the RDB of the S1 subunit of the Spike protein (S protein) of SARS-Cov-2. It is indicated to determine and monitor the immune response of individual who have been vaccinated and/or have/had COVID-19. These tests were performed by the COVID-19 Diagnostic Support Unit Laboratory in FIOCRUZ.

A subset of 50 participants underwent the plaque reduction neutralisation test (PRNT) for immunogenicity analysis pre 3rd dose and 28 days after. The test aimed to detect anti-SARS-CoV-2 neutralising antibodies at a Biosafety level 3 facility by using a model of infection in Vero cells, as previously described.^8^ The expressed titres of neutralising antibodies were determined by their capacity to neutralise 50% of plaque number. The PRNT was conducted by Nacional Reference Laboratory for Respiratory Viruses/Instituto Oswaldo Cruz (IOC)/FIOCRUZ.

*Statistical analysis* - In assessing immunogenicity, the study compared the GMT of anti-S antibodies, 28 days after administering the 3rd dose of the Covid-19 (recombinant) vaccine, given four to eight months after the second dose with an eight-week interval between the first two doses, to the GMT with 12-week interval between the first two doses. The calculated GMT of antibodies after Covid-19 (recombinant) vaccination, based on Flaxman et al. results,^6^ was 3495 with a 95% confidence interval (CI) (2833; 4312). The equivalence margin for these adopted titres ratio is 0.77 to 1.3, equivalent to -0.114 to 0.114 considering the difference in logarithms, which was arbitrarily defined to represent the acceptable variation of GMT across comparison groups, given the precision of antibody titres and the implications in disease protection. The calculation to determine the sample size assumed an equivalence design with 90% statistical power, equivalence margin for the ratio between 0.77 and 1.3, and a significance level of 5%, resulting in 265 participants per group.

To assess equivalence the study proposed calculating the ratio of GMT 28 days after the third dose, along with their respective 95% CI. The GMT for participants who received the second dose eight weeks after the first was considered equivalent to the GMT for participants with a 12-week interval between the first two doses if the 95% CI of the ratio of GMT is between 0.77 and 1.3.

Statistical analyses were conducted with the software IBM SPSS Statistics 20.0 (IBM Corp. Released, 2011).

*Ethics* - The procedures followed were in accordance with Resolution 466 of December 12, 2021, from the National Health Council regarding research involving human subjects and national regulations, and in accordance with the Good Clinical Practices guidelines outlined in the Americas Document and the Good Clinical Practice Manual of the International Conference on Harmonisation.

The study was approved by the research ethics committee at Instituto Nacional de Infectologia Evandro Chagas (INI/FIOCRUZ) and registered in ClinicalTrials.gov (NCT05142488). An informed consent was obtained after the nature and possible consequences of the study had been fully explained to the participants.

## RESULTS

Despite the field team’s efforts, the eight-week group had fewer participants (177) than calculated sample size (265). The recruitment of participants took place from November 2021 to May 2022. Challenge included a significant number of volunteers with suspected Covid-19 symptoms due to Omicron variant outbreak in Rio de Janeiro, and the fewer number of people vaccinated with the interval of eight weeks between the first and second dose. Various strategies, such as visits and surveys were employed, but obstacles like unsuccessful contacts and participants not meeting including criteria contributed to the reduced enrolment in the eight-week group.

*Demographic characteristics of the sample* - The participants with an average age of 40.1 years, exhibited a balanced gender distribution with 51.6 % male and 48.4% female. Among them, half identified as white (51%), and the average household size was 2.9 individuals. These characteristics in general, had small differences in the distribution in the comparisons groups (Table I).

**TABLE I:** Distribution of participants regarding demographic data

Demographic data	Eight weeks		12 weeks		Total	
	N	%	N	%	N	%
Sex						
Female	79	44,6	160	50,5	239	48,4
Male	98	55,4	157	49,5	255	51,6
Total	177	100,0	317	100,0	494	100,0
Race						
White	87	49,2	165	52,1	252	51,0
Black	18	10,2	59	18,6	77	15,6
Brown	72	40,7	93	29,3	165	33,4
Yellow	0	0,0	0	0,0	0	0,0
Indigenous	0	0,0	0	0,0	0	0,0
Total	177	100,0	317	100,0	494	100,0
Age group						
18 - 28	29	16,4	51	16,1	80	16,2
29 - 38	59	33,3	74	23,3	133	26,9
39 - 48	57	32,2	113	35,6	170	34,4
49 - 58	30	16,9	64	20,2	94	19,0
59 - 68	2	1,1	15	4,7	17	3,4
69 - 79	0	0,0	0	0,0	0	0,0
Above 79	0	0,0	0	0,0	0	0,0
Total	177	100,0	317	100,0	494	100,0
Age (years)						
Minimum	18		19		18	
Maximum	60		63		63	
Median	39		41		40	
Mean	38,9		40,8		40,1	
Standart deviation	9,4		10,6		10,2	
Number of people living in the same household						
Minimum	1		0		0	
Maximum	8		7		8	
Median	3		3		3	
Mean	2,9		2,9		2,9	
Standart deviation	1,2		1,2		1,2

*Immunogenicity* - The geometric mean titres increased after the third dose in both study groups compared to pre-vaccination titres by at least three times (Table II), with similar but slightly higher increases in the eight-week group. Values remained above pre-third doses levels at the three, six, and 12-month collections. Throughout all time intervals, seropositivity remained stable (Table III).

**TABLE II:** Geometric mean titres (GMT) of coronavirus disease 2019 (Covid-19) IgG antibodies (Elisa) and ratio of GMT post (variable timing) and pre-booster by study group

Collection time	Group		Ratio (Post/Pre)		CI (95% for the ratio)	
	Eight weeks	12 weeks	Eight weeks	12 weeks	Eight weeks	12 weeks
Pre 3rd dose	1453.5	1712.1	-	-	-	-
28 days post 3rd dose	5529.9	5425.4	3.8	3.2	(2.6; 5.6)	(2.4; 4.3)
Three months post 3rd dose	5546.2	5614.0	3.8	3.3	(2.6; 5.6)	(2.4; 4.4)
Six months post 3rd dose	6371.8	5423.0	4.4	3.2	(3; 6.4)	(2.4; 4.2)
12 months post 3rd dose	5926.4	6459.2	4.1	3.8	(2.8; 5.9)	(2.9; 5)

**TABLE III:** Distribution of research participants according to qualitative collection results by group and collection time

Collection time/ Qualitative result	Group			
	Eight weeks		12 weeks	
	N	%	N	%
Pre 3rd dose				
Reagent	175	98,9	315	99,7
Non-reactive	2	1,1	1	0,3
28 days after 3rd dose				
Reagent	167	100,0	305	100,0
Non-reactive	0	0,0	0	0,0
Three months after 3rd dose				
Reagent	167	100,0	297	100,0
Non-reactive	0	0,0	0	0,0
Six months after 3rd dose				
Reagent	173	100,0	310	100,0
Non-reactive	0	0,0	0	0,0
12 months after 3rd dose				
Reagent	169	100,0	308	100,0
Non-reactive	0	0,0	0	0,0

Non-reactive: < 50.0 AU/mL; Reagent: ≥ 50.0 AU/mL.

However, when considering the strict protocol premises regarding the critical limits of the 95% CI for the GMT (0.77:1.3), and the achieved result (0.75:1.2), it must be acknowledged that the conclusion would lean towards non-equivalence for the study’s primary outcome (Table IV). GMT ratios did not differ substantially across age strata (Table V), but the study sample size did not allow assessment of equivalence within age groups. Individuals aged 60 years or more, started and sustained higher GMT compared to younger participants.

**TABLE IV:** Ratio of geometric mean titres (GMT) of the coronavirus disease 2019 (Covid-19) (recombinant) vaccine by groups and collection time

Collection time	Group		Ratio	CI (95%)
	Eight weeks	12 weeks		
Pre 3rd dose	1453.5	1712.1	1.2	(0.78; 1.77)
28 days post 3rd dose	5529.9	5425.4	1.0	(0.75; 1.29)
Three months post 3rd dose	5546.2	5632.8	1.0	(0.76; 1.35)
Six months post 3rd dose	6077.9	4892.7	0.8	(0.6; 1.08)
12 months post 3rd dose	5276.6	5709.7	1.1	(0.79; 1.48)

**TABLE V:** Ratio of geometric mean titres (GMT) of the coronavirus disease 2019 (Covid-19) (recombinant) vaccine among groups over time of collection

Collection time and age group	Group		Ratio	CI (95%)
	Eight weeks	12 weeks		
< 40 years				
Pre 3rd dose	1715.6	1927.3	1.1	(0.67; 1.89)
28 days post 3rd dose	5706.8	5243.1	0.9	(0.64; 1.31)
Three months post 3rd dose	4970.0	5133.3	1.0	(0.73; 1.47)
Six months post 3rd dose	5182.1	4870.2	0.9	(0.67; 1.32)
12 months post 3rd dose	5112.5	5742.2	1.1	(0.83; 1.53)
40 to 60 years				
Pre 3rd dose	1204.7	1482.1	1.2	(0.66; 2.31)
28 days post 3rd dose	5343.8	5437.1	1.0	(0.66; 1.56)
Three months post 3rd dose	6231.3	5744.5	0.9	(0.59; 1.44)
Six months post 3rd dose	8014.4	5756.8	0.7	(0.49; 1.05)
12 months post 3rd dose	6985.0	7003.5	1.0	(0.71; 1.41)
> 60 years				
Pre 3rd dose	-	3619.7	-	-
28 days post 3rd dose	-	8824.1	-	-
Three months post 3rd dose	-	13838.0	-	-
Six months post 3rd dose	-	10111.0	-	-
12 months post 3rd dose	-	9642.6	-	-

Note: There are no participants aged over 60 who received the second dose eight weeks after the first dose.

The response to the different vaccine serotypes tested (Delta and Omicron BA.1 variants) were similar in both groups, with more significant increase for the Delta variant (three times) than for Omicron (two times) in the two tested groups (Table VI). It’s important to point out that both pre-3rd dose collections (11/9/2021 to 5/5/2022) and post-3rd dose collections (12/7/2021 to 6/27/2022) were conducted during the circulation period of the Delta and Omicron variants.^9^

**TABLE VI:** Ratio of geometric mean titres (GMT) of plaque reduction neutralisation test (PRNT) against isolated Delta and Omicron before and after 3rd dose per group

Mediam geometric titre	Group		Ratio (Post/Pre)		CI (95%) for the ratio	
	Eight weeks	12 weeks	Eight weeks	12 weeks	Eight weeks	12 weeks
PRTN Delta						
Pre 3rd dose	30.3	30.3	3.0	3.2	(1.4; 6.7)	(1.4; 7.2)
Post 3rd dose	91.9	97.1				
PRNT Omicron						
Pre 3rd dose	13.0	10.6	1.8	2.2	(0.9; 3.7)	(1; 4.8)
Post 3rd dose	23.9	23.6				

*Reactogenicity* - In the safety and reactogenicity analysis, it was found that there was no statistically significant difference between the groups regarding the frequency of all adverse events, both local and systemic (Table VII). The predominant local adverse events included local pain (72.2%) and oedema (20.5%), while the prevailing systemic events allegedly associated with vaccination were headache (44.3%) and drowsiness (37.4%).

**TABLE VII:** Distribution of participants regarding the types of systemic events allegedly associated with vaccination

Type of solicited adverse event	Eight weeks		12 weeks		Total		p-value
	Number of participants	%	Number of participants	%	Number of participants	%	
Local	34	19.8	54	17.6	88	18.4	0.612
Sistemic	16	9.3	21	6.8	37	7.7	
Local and Sistemic	98	57.0	178	58.0	276	57.6	
Total	148	86.0	253	82.4	401	83.7	

## DISCUSSION

This is a homologous equivalence study of immunogenicity after the third dose of the Covid-19 recombinant vaccine with an interval of six months after the second dose, comparing the interval of eight and 12 weeks between the first two doses. As a result, on the homologous booster of the Astra-Zeneca/Fiocruz vaccine revealed significant findings regarding the immune response. The booster dose was followed by strong immune responses 28 days after vaccination, and antibody levels remained high, at least until the latest tests 12 months after booster. The pattern was similar in both groups defined by interval between the first and second doses, but GMT ratios and 95% CI were not conclusive of equivalence. GMT was also similar in individuals aged less than 40 years and those 40-60 years old. The elderly showed a substantially response to the booster dose. The reduction in intervals by the Ministry of Health resulted in increased vaccine coverage and protection. Both groups maintained a robust immune response after the third dose.

Immunogenicity studies have major importance, as there are no well-established serological correlates of protection. Recent systematic reviews and meta-analysis were performed to understand the immune protection against infection. Alborz Rahmani and colleagues reviewed studies with viral vector and messenger ribonucleic acid vaccines, and the findings suggest that although a specific threshold for protection against different SARS-CoV-2 variants is yet not determined, higher binding antibody levels are associated with stronger protective effects.^10^ Sylvia Mink and colleagues pointed that protective threshold could guide booster vaccination recommendations, but verification in separate cohort is necessary.^11^

In previous studies, it has been noted that heterologous regimens elicit a potent antibody response.^12^ ^,^ ^13^ Our evaluation showed strong immune response in a homologous regimen sustained for 12 months, demonstrating the effectiveness of the homologous third dose. The pandemic and the ongoing viral circulation could also partly explain the sustained high titres up to a year after the booster dose.

In line with prior research,^14^ ^,^ ^15^ analysis of serum neutralisation tests for Delta and Omicron variants showed a more effective response against Delta than Omicron variant (BA.1) in both interval groups, with a note on the small sample size (50 participants).

The study faced limitations such as due to the pandemic context and lacked a comparison group with an alternative vaccine, impacting the ability to fully contextualise immunogenicity.

Overall, the results indicated the advantage of additional booster doses, consistent with the occurrence of Covid-19 cases among individuals who had been fully vaccinated. In fact, periodic revaccination has been repeated to keep the epidemic under control. The wide distribution and local production of the vaccine at Bio-Manguinhos played a crucial role in controlling the pandemic in Brazil.
